# COVID-19 Severity and Thrombo-Inflammatory Response Linked to Ethnicity

**DOI:** 10.3390/biomedicines10102549

**Published:** 2022-10-12

**Authors:** Beate Heissig, Yousef Salama, Roman Iakoubov, Joerg Janne Vehreschild, Ricardo Rios, Tatiane Nogueira, Maria J. G. T. Vehreschild, Melanie Stecher, Hirotake Mori, Julia Lanznaster, Eisuke Adachi, Carolin Jakob, Yoko Tabe, Maria Ruethrich, Stefan Borgmann, Toshio Naito, Kai Wille, Simon Valenti, Martin Hower, Nobutaka Hattori, Siegbert Rieg, Tetsutaro Nagaoka, Bjoern-Erik Jensen, Hiroshi Yotsuyanagi, Bernd Hertenstein, Hideoki Ogawa, Christoph Wyen, Eiki Kominami, Christoph Roemmele, Satoshi Takahashi, Jan Rupp, Kazuhisa Takahashi, Frank Hanses, Koichi Hattori, on behalf of the LEOSS Study Group

**Affiliations:** 1 School of Medicine, Juntendo University, 2-1-1 Hongo, Bunkyo-Ku, Tokyo 113-8421, Japan; 2 An-Najah Center for Cancer and Stem Cell Research, Faculty of Medicine and Health Sciences, An-Najah National University, P.O. Box 7, Nablus 99900800, Palestine; 3 Department of Internal Medicine II, University Hospital Rechts der Isar, School of Medicine, Technical University, 81675 Munich, Germany; 4 Medical Department II, University Hospital of Frankfurt, 60590 Frankfurt, Germany; 5 Institute of Computing, Federal University of Bahia, Salvador 40110060, Brazil; 6 Department of Internal Medicine, Infectious Diseases, University Hospital Frankfurt, Goethe University Frankfurt, 60590 Frankfurt am Main, Germany; 7 Department I for Internal Medicine, University Hospital of Cologne, University of Cologne, 50937 Cologne, Germany; 8 German Center for Infection Research (DZIF), Partner-Site Bonn-Cologne, 50937 Cologne, Germany; 9 Klinikum Passau, 94032 Passau, Germany; 10 IMSUT Hospital of The Institute of Medical Science, The University of Tokyo, Tokyo 108-8639, Japan; 11 Universitaetsklinikum, 07747 Jena, Germany; 12 Ingolstadt Hospital, 85049 Ingolstadt, Germany; 13 Johannes Wesling Klinikum Minden, Ruhr-Universitaet, 44801 Bochum, Germany; 14 Klinikum Dortmund gGmbH, Hospital of University Witten/Herdecke, 44137 Dortmund, Germany; 15 Universitaetsklinikum, 79106 Freiburg, Germany; 16 Universitaetsklinikum, 40225 Duesseldorf, Germany; 17 Klinikum Bremen-Mitte, 28205 Bremen, Germany; 18 Praxis am Ebertplatz Koeln, 50668 Köln, Germany; 19 Internal Medicine III—Gastroenterology and Infectious Diseases, University Hospital of Augsburg, 86156 Augsburg, Germany; 20 Department of Infectious Diseases and Microbiology, University Hospital Schleswig-Holstein/Campus Luebeck, 23538 Luebeck, Germany; 21 Emergency Department and Department for Infectious Diseases and Infection Control, University Hospital Regensburg, 93053 Regensburg, Germany

**Keywords:** COVID-19 disease severity, cardiovascular disease, comorbidities, coagulation, inflammation, PSM, biomarker, ethnicity, race

## Abstract

Although there is strong evidence that SARS-CoV-2 infection is associated with adverse outcomes in certain ethnic groups, the association of disease severity and risk factors such as comorbidities and biomarkers with racial disparities remains undefined. This retrospective study between March 2020 and February 2021 explores COVID-19 risk factors as predictors for patients’ disease progression through country comparison. Disease severity predictors in Germany and Japan were cardiovascular-associated comorbidities, dementia, and age. We adjusted age, sex, body mass index, and history of cardiovascular disease comorbidity in the country cohorts using a propensity score matching (PSM) technique to reduce the influence of differences in sample size and the surprisingly young, lean Japanese cohort. Analysis of the 170 PSM pairs confirmed that 65.29% of German and 85.29% of Japanese patients were in the uncomplicated phase. More German than Japanese patients were admitted in the complicated and critical phase. Ethnic differences were identified in patients without cardiovascular comorbidities. Japanese patients in the uncomplicated phase presented a suppressed inflammatory response and coagulopathy with hypocoagulation. In contrast, German patients exhibited a hyperactive inflammatory response and coagulopathy with hypercoagulation. These differences were less pronounced in patients in the complicated phase or with cardiovascular diseases. Coagulation/fibrinolysis-associated biomarkers rather than inflammatory-related biomarkers predicted disease severity in patients with cardiovascular comorbidities: platelet counts were associated with severe illness in German patients. In contrast, high D-dimer and fibrinogen levels predicted disease severity in Japanese patients. Our comparative study indicates that ethnicity influences COVID-19-associated biomarker expression linked to the inflammatory and coagulation (thrombo-inflammatory) response. Future studies will be necessary to determine whether these differences contributed to the less severe disease progression observed in Japanese COVID-19 patients compared with those in Germany.

## 1. Introduction

Clinical manifestations caused by the severe acute respiratory syndrome-coronavirus-2 (SARS-CoV-2) range from mild respiratory symptoms to pneumonia and, in extreme cases, multiorgan failure or even death.

Identifying simple, reliable risk factors such as comorbidities and blood biomarkers that could distinguish at the time of diagnosis those patients with the highest risk of fatal illness is essential for managing and treating coronavirus disease 2019 (COVID-19) patients. Hypertension, diabetes, and heart disease (called here “cardiovascular comorbidities”) are associated with more severe diseases in COVID-19 patients [1,2,3]. Retrospective cohort studies have reported that age, sex, ethnicity, and socio-economical parameters contribute to severe disease outcomes [4,5].

Comparative studies on the significance of ethnicity for understanding risk factors that could predict disease severity in COVID-19 patients have rarely been conducted. Studies suggest that COVID-19-associated coagulopathy and inflammatory parameters such as CRP levels are survival determinants [6,7]. Ethnicity is an essential determining factor in the coagulation response (thrombotic risk), with Asians (Japanese) having a much lower thrombotic risk than their Western counterparts, including Germans [8]. Japanese patients with cardiovascular diseases have a low mortality rate. National epidemiological COVID-19 studies have been carried out in Germany and Japan [2,9].

Germany and Japan are both developed countries with excellent health care capacities. Despite mass testing and strict social distancing measures, Germany’s infection rates and COVID-19-associated death rates were high throughout the pandemic. Germany reported 29,245 cases (and Japan 3416 cases) of COVID-19 per one million individuals until 28 February 2021 (https://www.worldometers.info/coronavirus/#countries, accessed on 9 September 2022). In contrast, Japan’s slow increase in daily new infections and its low death rate during the first waves of the pandemic surprised the world.

This retrospective study explores risk factors such as comorbidities for patients’ disease progression through country comparison and describes coagulation-associated and inflammation-associated biomarkers that could predict disease progression. Japan and Germany were chosen for the analysis because previous non-COVID-19 studies suggested that typical risk factors such as cardiovascular disease and thrombosis risk differ between both countries.

## 2. Results

In the study period between March 2020 and February 2021, 6149 SARS-CoV-2-positive German nationals were registered in the Lean European Open Survey on SARS-CoV-2 (LEOSS) registry, and 176 Japanese SARS-CoV-2-positive patients were recruited at two major hospitals. Larger data sets of Japanese COVID-19 patients were not available at the time of analysis.

An essential challenge in analyzing retrospective cohorts is the differences in absolute numbers of instances from the groups under scrutiny. Such differences may affect results, possibly emphasizing spurious relationships and affecting further conclusions in situations where new data collection is not feasible, as in the present study. Adopting well-established approaches is a necessary requirement to reduce the bias caused by differences in absolute numbers in specific covariates. Here, a two-step approach was chosen to identify national differences in COVID-19 risk factors between the German and Japanese cohorts that could predict disease severity.

As a first step, we examined basic clinical parameters that indicate disease severity in COVID-19 patients in the national cohorts independently of each other. In the initial analysis, primary clinical and epidemiological parameters, including ethnicity, age, sex, body mass index (BMI), and comorbidities, were examined separately as predictors of severe illness in both national cohorts.

### 2.1. Milder COVID-19 Disease Course in Japanese Patients Than in German Patients

The German patient cohort was 78.9% Caucasian, 0.1% Hispanic/Latino, 2.7% Asian, 1.5% African/African American, and 16.8% unknown. The Japanese patient cohort was 97% people of Asian ethnicity.

The male sex was dominant in both countries (Table 1). However, Japanese COVID-19 patients were younger on average.

German patients were more often overweight (>25 kg/m^2^), while >50% of Japanese patients presented with an average BMI (Table 1).

Most patients were in the uncomplicated phase at diagnosis, with 69% in Germany and 84% in Japan. More German patients (25%) than Japanese patients (13%) were in the complicated phase, and 6% versus 3%, respectively, were in the critical phase at diagnosis (Table 1).

These data suggest that Japanese COVID-19 patients experienced a milder disease than Germans.

### 2.2. Cardiovascular Comorbidities Predict Severe Disease in the Japanese and German Cohorts

A prediction analysis was performed to determine if any basic clinical parameters predicted severe illness in both country cohorts separately. The investigation revealed that advanced age, male sex, and a high BMI were associated with more severe disease (complicated/severe phase) in the German cohort (Table 1). In contrast, only age correlated with disease severity in the Japanese cohort.

Patients with underlying comorbidities experience more severe COVID-19 [10]. Univariate analysis among Japanese and German patients at diagnosis revealed that cardiovascular comorbidities or dementia indicated a more severe disease in both cohorts (Table 2). Kidney disease, hemiplegia, cerebrovascular, vascular, gastrointestinal, or respiratory disease indicated severe disease only in the German cohort (Table 2). Cancer, immunosuppression, and liver disease were not indicators of severe disease in either country cohort (Table 2). These data suggest that underlying cardiovascular comorbidities predicted disease severity in both countries.

Kaplan–Meier curves using either death or in-hospital disease progression can be used to compare disease severity between countries. Due to the low number of deaths in the Japanese cohort, the time at which hospital patients progressed from the uncomplicated to the complicated phase (length of hospital stay, LOS) was used to estimate disease severity in the whole-patient cohorts. Japanese but not German patients with dementia had a shorter LOS (*p* = 0.039; *p* = 0.17; 95% CI; Supplementary Figure S1). Heart disease (n = 1049, *p* < 0.0001, *p* = 0.046), hypertension (*p* < 0.0001, *p* < 0.00034), and diabetes (*p* < 0.0001, *p* = 0.024) correlated with a shorter LOS and a severe disease in both the German and Japanese cohorts. Hemiplegia, kidney, cerebrovascular, vascular, gastrointestinal, or respiratory disease indicated severe disease in the German but not the Japanese cohort (Table 2). These data confirm that cardiovascular comorbidities predicted disease progression in both countries.

### 2.3. Fewer Japanese Suffer from Severe Illness According to Propensity Score Matched Data Sets

The initial study analysis performed in both national cohorts indicated striking differences in the disease severity (a milder disease course in Japanese patients), sample size, age, BMI, and sex, but suggested that cardiovascular comorbidities were a common risk factor for disease severity in both cohorts. Parameters such as BMI, age, or underlying preexisting diseases, such as a history of cardiovascular comorbidities, can impact biomarkers independent of the SARS-CoV-2 infection.

**Figure 1:**
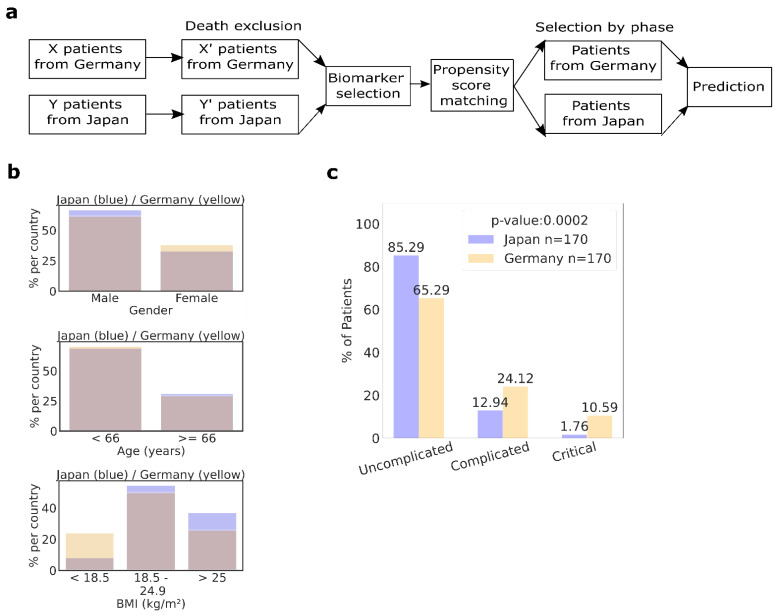
Baseline characteristics of German and Japanese COVID-19 cohorts after propensity score matching (PSM). (**a**) Scheme of the data analysis using PSM. (**b**) PSM for German and Japanese patient characteristics (n = 170/group): Adjusted variables were sex (top panel), age (middle panel), and BMI (lower panel). Blue indicates the excess found in Japanese patients. Yellow indicates excess found in German patients. (**c**) Distribution of patients in indicated clinical phases at diagnosis of PSM patients. *p* < 0.05 was regarded as significant.

Further analysis was necessary to reduce the influence of bias by applying a propensity score match (PSM) technique to simulate randomization and confound variables to better estimate severe disease outcomes. We adjusted age, sex, BMI, and history of cardiovascular disease comorbidity using a PSM pair technique to reduce bias due to differences in sample size (176 versus 6149), patient age, BMI, or comorbidity presence (the overall younger and leaner Japanese cohort had fewer comorbidities) (Figure 1a,b). After modeling the cohort using PSM, we were able to balance the Japanese and German patients, thus preparing our data for the predictive analyses.

Analysis of the 170 PSM pairs confirmed that 65.29% of German patients and 85.29% of Japanese patients were in the uncomplicated phase. Furthermore, 24.12% of German patients compared with 12.94% of Japanese patients were admitted in the complicated phase, and 10.59% versus 1.76%, respectively, in the critical phase (Figure 1c). Importantly, the PSM analysis reconfirmed our initial data, demonstrating that a lower percentage of Japanese suffered from severe illness.

### 2.4. Japanese, but Not Germans, without Cardiovascular Comorbidities at Diagnosis, Presented a Milder Inflammatory Response

**Figure 2:**
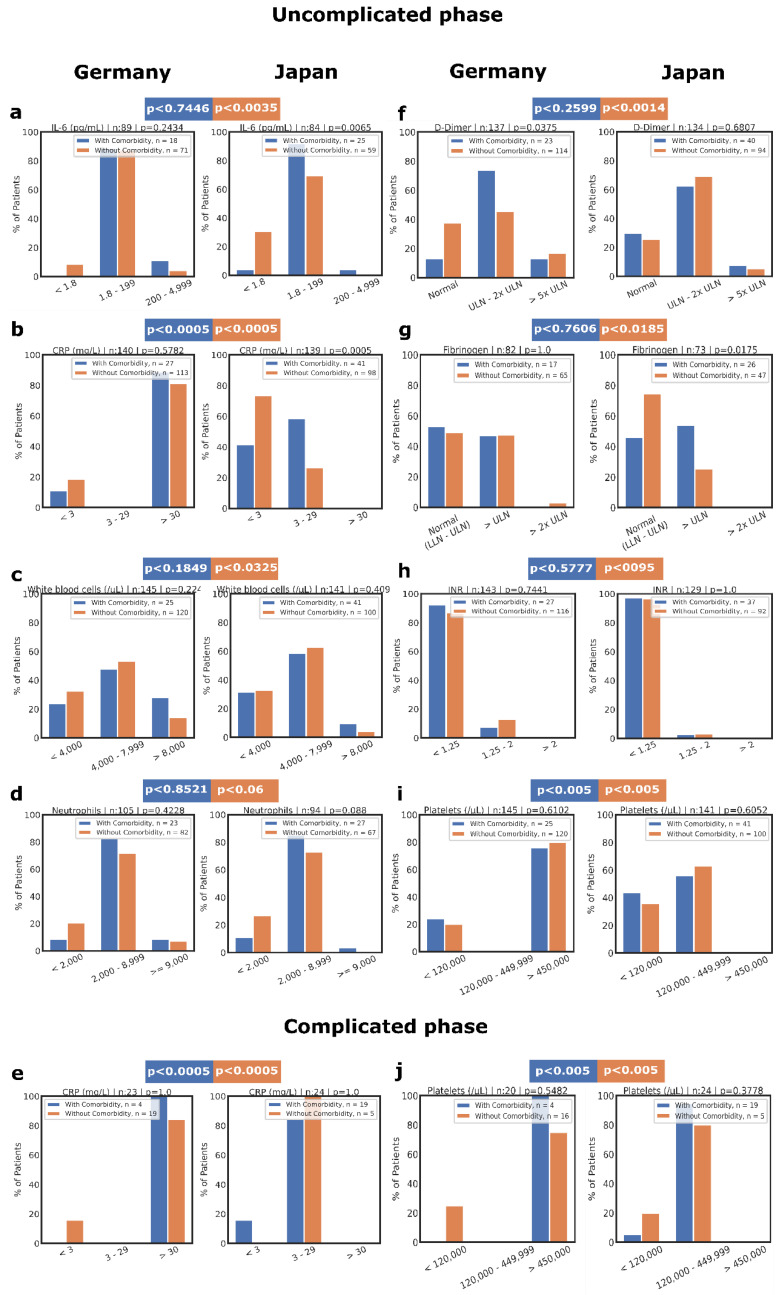
Biomarker comparison of PSM pairs of Japanese and German COVID-19 patients depending on the clinical phase and presence of cardiovascular diseases. Inflammatory biomarkers, including IL-6 (**a**) and CRP levels (**b**,**i**), or WBC (**c**) and neutrophil (**d**) counts, were plotted at the time of diagnosis of COVID-19 in patients in the uncomplicated or complicated phases. In addition, coagulation-associated biomarkers comprising levels of D-dimer (**f**), fibrinogen (**g**), or international normalized ratio (INR) (**h**), as well as platelet counts (**i**,**j**), were plotted at the time of diagnosis. (**e**,**f**) Patients in the complicated and critical phases were combined as the complicated phase. (**e**) Inflammatory biomarker CRP and (**f**) coagulation-associated biomarker platelet counts of patients in complicated phase. X^2^ or Fischer’s exact test determined the *p* values. *p* < 0.05 was regarded as significant.

Blood biomarkers are needed for early risk stratification and improved inpatient management of COVID-19 patients. We hypothesized that country-specific differences in disease severity might be rooted in differences in the inflammation and coagulation response. Compared with matched German PSM patients, Japanese patients in the uncomplicated phase, without cardiovascular comorbidities at diagnosis, presented a suppressed inflammatory response with lower interleukin-6 (IL-6) or C-reactive protein (CRP) levels and white blood cell (WBC) counts, but without reduced neutrophil counts (Figure 2a–d). These data indicate that Japanese but not German COVID-19 patients without cardiovascular comorbidities responded with a milder inflammatory response.

In contrast, German and Japanese patients with cardiovascular disease showed a similar inflammatory response, except for CRP (Figure S2a–f). Lower CRP persisted in Japanese patients with cardiovascular disease (*p* < 0.06) in the uncomplicated and complicated phases (Figure 2e). These data suggest that an underlying cardiovascular disease was linked with a more robust inflammatory response in COVID-19 patients independent of their ethnic background.

### 2.5. Coagulopathy with Hypocoagulation/Hyperfibrinolysis in Japanese Patients and Coagulopathy with Hypercoagulation in German Patients without Comorbidities

During inflammation, the coagulation and fibrinolytic system is activated [10]. Next, we analyzed coagulation/fibrinolysis-associated factors in the PSM patients. In the uncomplicated phase, Japanese patients without cardiovascular disease showed increases in D-dimers, mostly normal fibrinogen or INR (international normalized ratio) levels, and average platelet counts (65% in Japanese and 0% in German patients; Figure 2f–i). On the other hand, German patients showed abnormal platelet counts (Figure 2i) and had moderate increases in D-dimers, but high fibrinogen levels. These results indicate that Japanese COVID-19 patients in the uncomplicated phase showed a biomarker profile of coagulopathy with hypocoagulation/hyperfibrinolysis. In contrast, coagulopathy with hypercoagulation and hypofibrinolysis characterized the response in German COVID-19 patients (Supplementary Figure S2g).

### 2.6. No Racial Differences in the Coagulation/Fibrinolysis Response in Patients with Cardiovascular Comorbidities

Patients with underlying cardiovascular disorders were analyzed to determine whether the presence of cardiovascular comorbidities alters the biomarker profile. Although patients without cardiovascular comorbidities showed a country-specific coagulation/fibrinolysis profile, no country-specific differences in D-dimer, fibrinogen, or INR levels were observed in patients with underlying cardiovascular comorbidities (Figure 2f–h). Surprisingly, Japanese patients mainly had average thrombocyte counts, even those in the complicated phase and those with underlying cardiovascular diseases (Figure 2i,j). Furthermore, other coagulation/fibrinolysis-associated biomarker levels (D-dimer, fibrinogen, and INR) in the complicated phase were not influenced by an underlying cardiovascular disease, nor did they show country-specific differences (Supplementary Figure S2d–f).

### 2.7. Thrombo-Inflammatory Biomarkers as Predictors of Severe COVID-19 Differ between Country Cohorts

The quantitative differences in inflammatory and coagulation/fibrinolysis biomarkers at the time of admission prompted us to evaluate whether they were indicative of the future clinical course and a predictor for disease severity. The inflammatory markers IL-6, CRP, WBC, and neutrophils did not predict severe illness in the matched Japanese and German PSM cardiovascular data set (Table 3).

In contrast, the coagulation/fibrinolysis-associated biomarker platelet counts predicted severe disease in German patients with cardiovascular comorbidities, while high D-dimer and fibrinogen levels predicted disease severity in Japanese patients (Table 4).

## 3. Discussion

Several studies have identified biomarkers and comorbidities associated with COVID-19 infection, yet few have compared COVID-19 patient cohorts with high versus low disease severity based on racial and ethnic differences. In this retrospective study of German and Japanese COVID-19 patients, age, BMI, sex, and cardiovascular disease were investigated to determine whether they are risk factors for severe COVID-19 [11,12,13,14].

The dominance of the male sex in both country cohorts agrees with previous reports. Sex differences, e.g., variations in hormone concentrations in males and females, might affect the immune response to microbes [13].

Although various comorbidities have been proposed as indicators of an increased risk for severe COVID-19, only cardiovascular diseases were common comorbidities in the German and Japanese patient cohorts, supporting previous reports [14,15,16,17].

Fewer Japanese COVID-19 patients were in the complicated/severe disease phase in the PSM-matched country cohorts. To understand which biomarkers are associated with severe COVID-19, we analyzed 170 German and Japanese patients that were PSM-matched for age, sex, BMI, and cardiovascular comorbidity. Inflammatory markers, such as IL-6, D-dimer, CRP, and/or ferritin, are believed to correlate directly with mortality in COVID-19 [18,19]. Inflammatory markers are strongly associated with critical illness and mortality in COVID-19 patients [20]. Our data suggest that IL-6 levels are low in Japanese patients, suggesting that most Japanese would not benefit from IL-6 receptor antibody tocilizumab treatment [21].

High CRP levels at admission were associated with respiratory support escalation and, therefore, the severity of the disease [19]. Increased circulating CRP levels correlate with an increased risk of multiple organ failure and death [22]. In contrast to the current study, in which CRP levels were lower in the Japanese compared with the German cohort, another study on the influence of ethnicity in COVID-19 patients revealed that CRP values in Asians/Indians and Hispanics were higher than in Whites [5]. Differences in the study design might be responsible for the contrasting results. While the study by Go et al. recruited patients of different ethnicity, they were all living in the USA, whereas the current study compared patients residing in their country of ethnic origin with those of another.

Our data demonstrate that coagulation/fibrinolysis biomarkers, but not CRP or IL-6 levels [23,24], indicate severe illness in both country cohorts. Severe COVID-19 is associated with coagulopathy of hypercoagulation and an increase in D-dimer levels [25]. Confirming data produced by Zhou et al., D-dimers were indicators for poor prognosis in Japanese COVID-19 patients [26]. Thrombocytopenia indicates a poor prognosis in SARS, MERS, and COVID-19 [27]. A meta-analysis showed that the most severe manifestations of COVID-19 were associated with lower platelet counts than those observed in milder forms of the disease [28]. Thrombocyte counts were the only predictors of severe illness in the German cohort.

In the uncomplicated phase, Japanese COVID-19 patients presented with a suppressed inflammatory response and coagulopathy with hypocoagulation. In contrast, German COVID-19 patients showed a strong inflammatory response and coagulopathy with hypercoagulation (Figure S2g). COVID-19-associated coagulopathy with hypercoagulation is characterized by prominent elevation of fibrinogen and D-dimer levels [29], and constitutes an independent biomarker for poor prognosis in COVID-19. Increased D-dimer levels in COVID-19 patients [3,30] likely reflect pulmonary vascular bed thrombosis or asymptomatic deep vein thrombosis [31].

Coagulopathy with hypocoagulation in Japanese COVID-19 patients could reduce the risk of thromboembolic events. Indeed, a retrospective study of 6202 Japanese COVID-19 patients found that the thrombosis risk in mild/moderate patients was low at 0.59% [29]. Higher thrombotic risk in Japanese patients was mainly reported in the complicated phase [30,32].

Ethnicity affects thrombotic risk, with a lower risk in Asians than in Caucasians [33,34,35]. The observation of the low incidence of thrombosis in Asia, independent of COVID-19 infection, has been supported by various studies. For example, the standardized and adjusted incidence of venous thromboembolism per 100,000 Californian adults was as low as 21 in individuals of Asian/Pacific Islander ethnicity, compared with 104 in Caucasians, 141 in African-Americans, and 55 in Hispanics [36].

Genetic alterations of coagulation factors are more widespread in Asia than in Western countries. For example, mutations in the vitamin K epoxide reductase complex 1 gene cause a deficiency in vitamin K-dependent clotting factors, increasing the sensitivity of Japanese patients to warfarin [37]. Similar genetic mutations are found in the fibrinogen gene [38]. Following a study by Long et al. [39], high fibrinogen and D-dimer levels predicted disease severity in Japanese but not German PSM patients. Future studies should address whether these genetic differences help protect the Japanese against adverse responses to SARS-CoV-2 infection.

The study’s limitations include using categorical biomarker values that might influence the accuracy of the Pearson analysis. Nevertheless, our results show that Japanese people show a demographic, comorbidity, and biomarker profile that could contribute to less severe COVID-19. Further studies are needed to establish whether the “natural” protection against SARS-CoV-2 infections observed in the Japanese population is rooted in differences in genetic and/or environmental factors.

In conclusion, cardiovascular comorbidities and age predicted severe illness in COVID-19 patients, independent of ethnic differences. The mainly Caucasian (German) and Asian (Japanese) cohorts presented with country-specific inflammation- and coagulation/fibrinolysis-associated biomarker profiles. These biomarkers served as reliable disease severity predictors only within the respective ethnic group.

## 4. Materials and Methods

### 4.1. Study Design, Participants, and Data Collection

The inclusion criteria in both country populations were adult patients older than 18 years, in- and outpatients, patients with a positive PCR or rapid antigen test for SARS-CoV-2, availability of basic medical information, including ethnicity, patient history, initial blood laboratory data, and outcome data. The study period spanned March 2020 to February 2021.

The German study population comprised adults registered in LEOSS. The Japanese study population included adults admitted to Juntendo University, Japan, or the Institute of Medical Science, the University of Tokyo (IMSUT), Japan. All study participants gave informed consent for the anonymized use of their clinical data.

We defined the clinical phases of COVID-19 at diagnosis as (1) uncomplicated (either asymptomatic or with symptoms of upper respiratory tract infection, fever, nausea, emesis, or diarrhea), (2) complicated (need for oxygen supplementation or clinically relevant increase in prior oxygen home therapy, partial pressure of oxygen (PaO_2_) at room air <70 mmHg, SO_2_ at room air <90%, new cardiac arrhythmia, new pericardial effusion >1 cm or new heart failure with pulmonary edema, congestive hepatopathy, or peripheral edema), and (3) critical (need for life-supporting therapy, such as mechanical ventilation, catecholamine dependence, life-threatening cardiac arrhythmia, liver failure with an INR >3.5, a qSOFA score > +2, or acute renal failure with the need for dialysis) [14] (see also https://leoss.net/statistics/, accessed 1 February 2021). Criteria for discharge were the absence of fever for at least three days, substantial improvement in both lungs, clinical remission of respiratory symptoms, and one negative PCR test result for SARS-CoV-2 RNA.

We sorted comorbidities according to the organ systems affected: heart disease (myocardial infarction, coronary disease, congestive heart failure), cerebrovascular comorbidity (stroke), liver disease (chronic liver disease, hepatitis, or cirrhosis), kidney disease (acute and chronic kidney disease), and immunosuppressive status (post organ transplantation, HIV, or collagen diseases).

Blood examinations included the coagulation/fibrinolysis factors D-dimer (≤1 ug/mL), INR (<1.25), fibrinogen (≤400 mg/dL), and platelet counts (120,000–450,000/uL) as well as the inflammatory factors CRP (≤3 mg/L), IL-6 (≤1.8 pg/mL), WBC (4000–8000/uL), and neutrophils (2000–8900/uL). Normal values are given in brackets.

### 4.2. Statistical Analysis

We compared the characteristics of SARS-CoV-2-positive patients according to their country of origin (German versus Japanese). The basis of the LEOSS data is an individual scientific use file (SUF), generated after a complex anonymization procedure, as described earlier [40].

We presented continuous and categorical variables as median and n (%), respectively. Before predicting the patients’ clinical phases, we organized comorbidities and biomarkers as categorical variables (yes versus no). We performed the prediction analysis by considering the clinical phase, and estimated independence between variables using the experimental setup presented in Supplementary Figure S2h [41]. First, we calculated a contingency matrix by counting the frequencies of patients in different clinical phases and specific variable intervals. Then, the statistical dependence test was performed based on the number of intervals and the obtained frequencies. We considered Fisher’s exact test if the contingency table had a 2 × 2 shape (two-variable intervals). We chose the appropriate test when tables with more than two intervals were analyzed, i.e., we executed the X^2^ test when the frequencies in each cell were at least five. However, in the case of analyzing tables with more intervals and some frequencies below five, Fischer’s exact test simulation was performed [42] using the Monte Carlo approach, available in the R statistical package 3.6.3. This package was used to calculate the Pearson correlation test of the numerical values for all biomarkers.

Survival analyses/Kaplan–Meier curves for the time intervals until disease progression were produced. Hospital patients in the uncomplicated phase at diagnosis were selected when we found data on the disease phase in the follow-up or discharge from the hospital within 50 days. It was necessary that patients had spent at least one day in the hospital, did not die during hospitalization, and were discharged from the hospital before the end of the study. We performed survival analysis using as the outcome variable time spent in hospital until the disease progressed from the uncomplicated to the complicated phase. For the survival analysis, patients were segregated into the existence (yes) or the absence (no) of comorbidity groups.

### 4.3. PSM Analysis

Patients were matched 1:1, adjusting age, sex, body-mass index, history of hypertension, heart disease, and diabetes, and randomly sorted using the nearest-neighbor strategy with acceptable distance (caliper) of propensity scores [43]. We assessed matching quality using standardized mean differences for outcome analysis (biomarker analysis).

A patient was judged to be without cardiovascular comorbidity when there was no cardiovascular comorbidity, but other comorbidities could have been recorded. Patient clinical outcomes were death or discharge from hospital. We imputed the mean value for patients with missing data for the BMI. *p* < 0.05 was regarded as significant.

## Figures and Tables

**Table 1:** **Univariate analysis of baseline clinical parameters as indicators for disease progression among Japanese and German patients at diagnosis.** SARS-CoV-2-positive patients from Japan and Germany on admission in the uncomplicated, complicated, or critical phase of COVID-19. Data are n (%). BMI = body mass index; *p*-values were calculated as appropriate by X^2^ or Fischer’s exact test.

	GERMANY					JAPAN				
	Total	Uncomplicated	Complicated	Critical	*p*-Value	Total	Uncomplicated	Complicated	Critical	*p*-Value
Clinical Phase										
	6059	4161.0 (69.0%)	1513.0(25.0%)	385.0 (6%)		174	147.0 (84.0%)	22.0 (13%)	5.0 (3.0%)	
Age										
<26 years	250	233.0 (93.2%)	10.0 (4.0%)	7.0 (2.8%)	0	15	15.0 (100.0%)	0.0 (0.0%)	0.0 (0.0%)	0.0005
26–35 years	447	394.0 (88.14%)	43.0 (9.62%)	10.0 (2.24%)		22	22.0 (100.0%)	0.0 (0.0%)	0.0 (0.0%)	
36–45 years	527	427.0 (81.02%)	85.0 (16.13%)	15.0 (2.85%)		24	24.0 (100.0%)	0.0 (0.0%)	0.0 (0.0%)	
46–55 years	924	654.0 (70.78%)	214.0 (23.16%)	56.0 (6.06%)		32	30.0 (93.75%)	2.0 (6.25%)	0.0 (0.0%)	
56–65 years	1112	761.0 (68.44%)	265.0 (23.83%)	86.0 (7.73%)		27	20.0 (74.07%)	5.0 (18.52%)	2.0 (7.41%)	
66–75 years	991	592.0 (59.74%)	292.0 (29.47%)	107.0 (10.8%)		29	23.0 (79.31%)	5.0 (17.24%)	1.0 (3.45%)	
76–85 years	1281	781.0 (60.97%)	422.0 (32.94%)	78.0 (6.09%)		21	11.0 (52.38%)	8.0 (38.1%)	2.0 (9.52%)	
>85 years	497	295.0 (59.36%)	177.0 (35.61%)	25.0 (5.03%)		4	2.0 (50.0%)	2.0 (50.0%)	0.0 (0.0%)	
Sex										
M	3496	2295.0 (65.65%)	928.0 (26.54%)	273.0 (7.81%)	0	116	95.0 (81.9%)	18.0 (15.52%)	3.0 (2.59%)	0.2668
F	2549	1855.0 (72.77%)	582.0 (22.83%)	112.0 (4.39%)		58	52.0 (89.66%)	4.0 (6.9%)	2.0 (3.45%)	
BMI										
<18.5 kg/m^2^	106	81.0 (76.42%)	19.0 (17.92%)	6.0 (5.66%)	0	14	14.0 (100.0%)	0.0 (0.0%)	0.0 (0.0%)	0.1044
18.5–24.9 kg/m^2^	1135	807.0 (71.1%)	275.0 (24.23%)	53.0 (4.67%)		93	73.0 (78.49%)	17.0 (18.28%)	3.0 (3.23%)	
25–29.9 kg/m^2^	1255	826.0 (65.82%)	350.0 (27.89%)	79.0 (6.29%)		52	47.0 (90.38%)	5.0 (9.62%)	0.0 (0.0%)	
30–34.9 kg/m^2^	672	419.0 (62.35%)	204.0 (30.36%)	49.0 (7.29%)		7	7.0 (100.0%)	0.0 (0.0%)	0.0 (0.0%)	
>34.9 kg/m^2^	381	211.0 (55.38%)	106.0 (27.82%)	64.0 (16.8%)		4	4.0 (100.0%)	0.0 (0.0%)	0.0 (0.0%)	

**Table 2:** Univariate analysis of comorbidities as indicators for disease progression among Japanese and German patients at diagnosis. Patients showing one specified comorbidity alone or a combination were enrolled in the yes group. Data indicate an association between comorbidity and higher odds of developing a more severe illness with a 95% confidence interval (CI). Data are n (% of patients in each group). *p* values were calculated by X^2^ test or Fischer’s exact test.

	GERMANY					JAPAN				
	Total	Uncomplicated	Complicated	Critical	*p*-Value	Total	Uncomplicated	Complicated	Critical	*p*-Value
**Heart**										
**Yes**	3355	2080.0 (62.0%)	1011.0 (30.13%)	264.0 (7.87%)	0	13	5.0 (38.46%)	8.0 (61.54%)	0.0 (0.0%)	0
**No**	2704	2081.0 (76.96%)	502.0 (18.57%)	121.0 (4.47%)		161	142.0 (88.2%)	14.0 (8.7%)	5.0 (3.11%)	
**Hypertension**										
**Yes**	2914	1800.0 (61.77%)	879.0 (30.16%)	235.0 (8.06%)	0	50	32.0 (64.0%)	18.0 (36.0%)	0.0 (0.0%)	0
**No**	3028	2301.0 (75.99%)	602.0 (19.88%)	125.0 (4.13%)		124	115.0 (92.74%)	4.0 (3.23%)	5.0 (4.03%)	
**Diabetes**										
**Yes**	1235	705.0 (57.09%)	417.0 (33.77%)	113.0 (9.15%)	0	27	18.0 (66.67%)	8.0 (29.63%)	1.0 (3.7%)	0.0127
**No**	4824	3456.0 (71.64%)	1096.0 (22.72%)	272.0 (5.64%)		147	129.0 (87.76%)	14.0 (9.52%)	4.0 (2.72%)	
**Dementia**										
**Yes**	503	295.0 (58.65%)	178.0 (35.39%)	30.0 (5.96%)	0	10	4.0 (40.0%)	5.0 (50.0%)	1.0 (10.0%)	0.0011
**No**	5369	3769.0 (70.2%)	1273.0 (23.71%)	327.0 (6.09%)		164	143.0 (87.2%)	17.0 (10.37%)	4.0 (2.44%)	
**Kidney**										
**Yes**	843	506.0 (60.02%)	285.0 (33.81%)	52.0 (6.17%)	0	12	8.0 (66.67%)	4.0 (33.33%)	0.0 (0.0%)	0.0944
**No**	5047	3567.0 (70.68%)	1175.0 (23.28%)	305.0 (6.04%)		162	139.0 (85.8%)	18.0 (11.11%)	5.0 (3.09%)	
**Hemiplegia**										
**Yes**	96	49.0 (51.04%)	36.0 (37.5%)	11.0 (11.46%)	0	4	2.0 (50.0%)	1.0 (25.0%)	1.0 (25.0%)	0.1142
**No**	5771	4012.0 (69.52%)	1412.0 (24.47%)	347.0 (6.01%)		170	145.0 (85.29%)	21.0 (12.35%)	4.0 (2.35%)	
**Cerebrovascular**										
**Yes**	505	302.0 (59.8%)	169.0 (33.47%)	34.0 (6.73%)	0	4	2.0 (50.0%)	2.0 (50.0%)	0.0 (0.0%)	0.1142
**No**	5554	3859.0 (69.48%)	1344.0 (24.2%)	351.0 (6.32%)		170	145.0 (85.29%)	20.0 (11.76%)	5.0 (2.94%)	
**Vascular**										
**Yes**	254	144.0 (56.69%)	91.0 (35.83%)	19.0 (7.48%)	0	4	2.0 (50.0%)	2.0 (50.0%)	0.0 (0.0%)	0.1142
**No**	5564	3893.0 (69.97%)	1337.0 (24.03%)	334.0 (6.0%)		170	145.0 (85.29%)	20.0 (11.76%)	5.0 (2.94%)	
**Respiratory**										
**Yes**	862	524.0 (60.79%)	270.0 (31.32%)	68.0 (7.89%)	0	4	3.0 (75.0%)	1.0 (25.0%)	0.0 (0.0%)	0.4938
**No**	5197	3637.0 (69.98%)	1243.0 (23.92%)	317.0 (6.1%)		170	144.0 (84.71%)	21.0 (12.35%)	5.0 (2.94%)	
**Immunosuppressive**										
**Yes**	393	268.0 (68.19%)	105.0 (26.72%)	20.0 (5.09%)	0.7526	10	7.0 (70.0%)	3.0 (30.0%)	0.0 (0.0%)	0.4387
**No**	5666	3893.0 (68.71%)	1408.0 (24.85%)	365.0 (6.44%)		118	94.0 (79.66%)	19.0 (16.1%)	5.0 (4.24%)	
**Cancer**										
**Yes**	766	509.0 (66.45%)	223.0 (29.11%)	34.0 (4.44%)	0.1333	12	11.0 (91.67%)	1.0 (8.33%)	0.0 (0.0%)	0.4583
**No**	5293	3652.0 (69.0%)	1290.0 (24.37%)	351.0 (6.63%)		116	90.0 (77.59%)	21.0 (18.1%)	5.0 (4.31%)	
**Liver**										
**Yes**	152	105.0 (69.08%)	35.0 (23.03%)	12.0 (7.89%)	0.9598	34	24.0 (70.59%)	9.0 (26.47%)	1.0 (2.94%)	0.2534
**No**	5907	4056.0 (68.66%)	1478.0 (25.02%)	373.0 (6.31%)		94	77.0 (81.91%)	13.0 (13.83%)	4.0 (4.26%)	
**Gastro**										
**Yes**	105	65.0 (61.9%)	30.0 (28.57%)	10.0 (9.52%)	0	0	0.0 (nan%)	0.0 (nan%)	0.0 (nan%)	1
**No**	5757	3993.0 (69.36%)	1418.0 (24.63%)	346.0 (6.01%)		174	147.0 (84.48%)	22.0 (12.64%)	5.0 (2.87%)	

**Table 3:** Effect of inflammation-associated biomarkers on clinical phase in propensity score-matched pairs of German and Japanese patients with cardiovascular disease.

Inflammation Biomarkers	GERMANY					JAPAN				
	Total	Uncomplicated	Complicated	Critical	*p*-Value	Total	Uncomplicated	Complicated	Critical	*p*-Value
**Interleukin-6**										
**<1.8**	0	0 (0%)	0 (0%)	0 (0%)	1.0000	1	1 (100.0%)	0 (0.0%)	0 (0.0%)	1.0000
**1.8–199**	19	16 (84.21%)	3 (15.79%)	0 (0.0%)		39	23 (58.97%)	16 (41.03%)	0 (0.0%)	
**200–4999**	2	2 (100.0%)	0 (0.0%)	0 (0.0%)		1	1 (100.0%)	0 (0.0%)	0 (0.0%)	
**CRP**										
**<3**	3	3 (100.0%)	0 (0.0%)	0 (0.0%)	1.0000	20	17 (85.0%)	3 (15.0%)	0 (0.0%)	0.0780
**3–29**	0	0 (0%)	0 (0%)	0 (0%)		40	24 (60.0%)	16 (40.0%)	0 (0.0%)	
**>30**	28	24 (85.71%)	3 (10.71%)	1 (3.57%)		0	0 (0%)	0 (0%)	0 (0%)	
**White blood cells**										
**<4000**	8	6 (75.0%)	1 (12.5%)	1 (12.5%)	0.2444	16	13 (81.25%)	3 (18.75%)	0 (0.0%)	0.2284
**4000–7999**	12	12 (100.0%)	0 (0.0%)	0 (0.0%)		36	24 (66.67%)	12 (33.33%)	0 (0.0%)	
**>8000**	8	7 (87.5%)	1 (12.5%)	0 (0.0%)		8	4 (50.0%)	4 (50.0%)	0 (0.0%)	
**Neutrophil**										
**<2000**	2	2 (100.0%)	0 (0.0%)	0 (0.0%)	1.0000	5	3 (60.0%)	2 (40.0%)	0 (0.0%)	1.0000
**2000–8999**	21	19 (90.48%)	2 (9.52%)	0 (0.0%)		39	23 (58.97%)	16 (41.03%)	0 (0.0%)	
**≥9000**	2	2 (100.0%)	0 (0.0%)	0 (0.0%)		2	1 (50.0%)	1 (50.0%)	0 (0.0%)	

Data are n (%). *p*-values were obtained by comparing the values of a biomarker within each country separately, considering the clinical phase at diagnosis. *p* values were calculated by X^2^ or Fischer’s exact test.

**Table 4:** Effect of coagulation/fibrinolysis-associated biomarkers on clinical phase in propensity score-matched pairs of German and Japanese patients with cardiovascular disease.

Thrombosis Biomarkers	GERMANY					JAPAN				
	Total	Uncomplicated	Complicated	Critical	*p*-Value	Total	Uncomplicated	Complicated	Critical	*p*-Value
**D-Dimers**										
**Normal**	4	3 (75.0%)	0 (0.0%)	1 (25.0%)	0.2624	12	12 (100.0%)	0 (0.0%)	0 (0.0%)	0.008
**ULN–2x ULN**	19	17 (89.47%)	2 (10.53%)	0 (0.0%)		39	25 (64.1%)	14 (35.9%)	0 (0.0%)	
**>5x ULN**	4	3 (75.0%)	1 (25.0%)	0 (0.0%)		8	3 (37.5%)	5 (62.5%)	0 (0.0%)	
**INR**										
**<1.25**	25	25 (100.0%)	0 (0.0%)	0 (0.0%)	0.099	54	36 (66.67%)	18 (33.33%)	0 (0.0%)	1.0000
**1.25–2**	3	2 (66.67%)	1 (33.33%)	0 (0.0%)		2	1 (50.0%)	1 (50.0%)	0 (0.0%)	
**>2**	0	0 (0%)	0 (0%)	0 (0%)		0	0 (0%)	0 (0%)	0 (0%)	
**Fibrinogen**										
**Normal (LLN–ULN)**	10	9 (90.0%)	0 (0.0%)	1 (10.0%)	1.0000	12	12 (100.0%)	0 (0.0%)	0 (0.0%)	0.0050
**>ULN**	9	8 (88.89%)	1 (11.11%)	0 (0.0%)		28	14 (50.0%)	14 (50.0%)	0 (0.0%)	
**>2x ULN**	0	0 (0%)	0 (0%)	0 (0%)		1	0 (0.0%)	1 (100.0%)	0 (0.0%)	
**Platelets**										
**<120,000**	19	18 (94.74%)	1 (5.26%)	0 (0.0%)	0.003	6	6 (100.0%)	0 (0.0%)	0 (0.0%)	1.0000
**120,000–449,999**	41	23 (56.1%)	18 (43.9%)	0 (0.0%)		0	0 (0%)	0 (0%)	0 (0%)	
**>450,000**	0	0 (0%)	0 (0%)	0 (0%)		23	19 (82.61%)	3 (13.04%)	1 (4.35%)	

Propensity score-matched data of coagulation/fibrinolysis-associated biomarkers are n (%). *p*-values were obtained by comparing the values of a biomarker within each country separately, considering the clinical phase at diagnosis. *p* values were calculated by X^2^ test or Fischer’s exact test.

## Data Availability

Access to SUF data should be discussed and voted on by the LEOSS governance. A public use file and a corresponding dashboard are available on the LEOSS website (https://leoss.net/data/, accessed on 9 September 2022). Access to the Japanese data requires approval from the ethics committees of both universities.

## Supplementary Materials

The following supporting information can be downloaded at: https://www.mdpi.com/article/10.3390/biomedicines10102549/s1, Figure S1: The in-hospital length of stay until disease progression (LOSUP) was used to measure COVID-19 severity and plotted in Kaplan–Meier curves. Curves were censored on day 50. The Kaplan–Meier estimates as of day 50 had a 95% CI. Kaplan–Meier survival curves as a function of the presence (with) or absence (without) of the indicated comorbidity in the whole-country cohorts (heart, total n = 1915/108, resp.; hypertension, total n = 1915/108, resp.; diabetes, total n = 1915/108, resp.; dementia, n = 1915/108, resp.). Comparison between both conditions in each plot with the indicated log-rank *p*-value. *p* < 0.05 was regarded as significant. Figure S2: Biomarker comparison of propensity score-matched pairs of German and Japanese patients in the complicated phase. (a–f) Biomarker comparison between Japan and Germany in COVID-19 patients in the complicated phase, whereby a patient was considered without cardiovascular comorbidity when only non-cardiovascular comorbidities could have been recorded. Serum levels of IL-6 (a), CRP (b), or neutrophil (c) counts in patients at diagnosis from Germany and Japan. Coagulation-associated parameters included levels of D-dimers (d), fibrinogen (e), and INR (f) in patients from Germany and Japan who presented in indicated phase at diagnosis. Patients in the complicated and critical phases were combined and summarized as complicated phases. P values were determined by X^2^ or Fischer’s exact test. *p* < 0.05 was regarded as significant. (g) Differential inflammation- and coagulation-associated responses of German and Japanese COVID-19 patients in the uncomplicated phase at diagnosis. Hyperinflammatory response and coagulopathy with hypercoagulation characterized patients from Germany in the uncomplicated phase. In contrast, patients in the uncomplicated phase in Japan showed a suppressed inflammatory response and coagulopathy with hypocoagulation. (h) Scheme of the data analysis. Comorbidities and biomarkers were organized as categorical variables (yes versus no) before the patients’ clinical phases within the whole population were predicted. The prediction analysis was performed by considering the clinical phase and estimated independence between variables using the indicated experimental setup.
