# Supplementary material for: COVID-19 Severity and Thrombo-Inflammatory Response Linked to Ethnicity
Source: Biomedicines. 2022 Oct 12;10(10):2549. doi: 10.3390/biomedicines10102549 (PMC9599040; doi:10.3390/biomedicines10102549)
Supplement: Supplementary file 1 [file biomedicines-10-02549-s001.zip › biomedicines-1936557-supplementary.pdf]

Supplementary Material
Heissig et al. Biomedicines

Article title

COVID-19 Severity and Thrombo-Inflammatory Response Linked to Ethnicity

journal name: **Biomedicines**

author names
Beate Heissig , Yousef Salama, Roman Iakoubov, Joerg Janne Vehreschild,
Ricardo Rios, Tatiane Nogueira, Maria J.G.T. Vehreschild,
Melanie Stecher, Hirotake Mori, Julia Lanznaster, Eisuke Adachi,
Carolin Jakob, Yoko Tabe, Maria Ruethrich, Stefan Borgmann,
Toshio Naito, Kai Wille, Simon Valenti, Martin Hower,
Nobutaka Hattori, Siegbert Rieg, Tetsutaro Nagaoka, Bjoern-Erik Jensen,
Hiroshi Yotsuyanagi, Bernd Hertenstein, Hideoki Ogawa,
Christoph Wyen, Eiki Kominami, Christoph Roemmele, Satoshi Takahashi,
Jan Rupp, Kazuhisa Takahashi, Frank Hanses, Koichi Hattori *,
on behalf of the LEOSS Study Group

Affiliation and email of corresponding author:
School of Medicine, Juntendo University, 2-1-1 Hongo, Bunkyo-Ku,
Tokyo 113-8421, Japan; khattori@juntendo.ac.jp

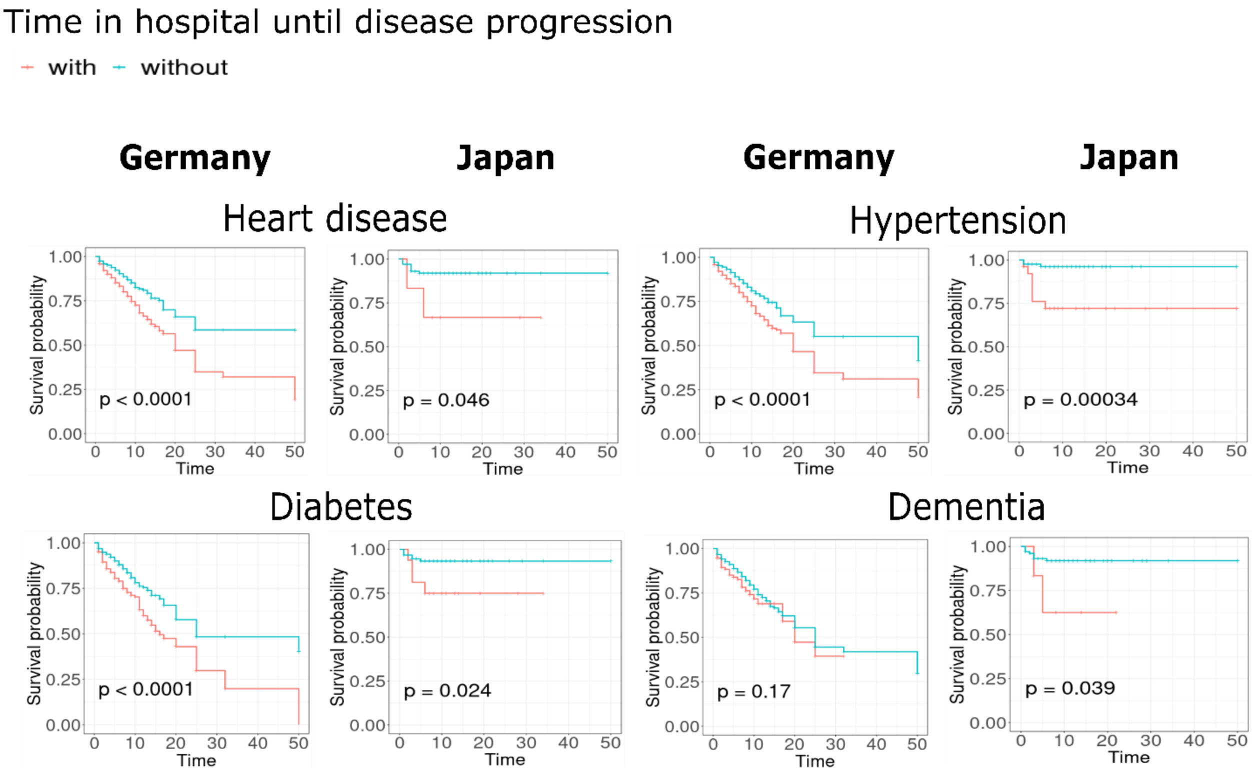

Supplementary Figure S1. The in-hospital Length of Stay Until disease Progression (LOSUP) was used to measure COVID-19 severity and plotted in Kaplan-Meier curves. Curves were censored on day 50. The Kaplan-Meier estimates as of day 50 had 95% CI. Kaplan-Meier survival curves as a function of the presence (with) or absence (without) of the indicated comorbidity in the whole country cohorts (heart, total n=1915/108, resp.; hypertension, total n=1915/108, resp.; diabetes, total n=1915/108, resp.; dementia, n=1915/108, resp.). Comparison between both conditions in each plot with the indicated log-rank p-value. P <0.05 was regarded as significant.

Supplementary Figure 2

Complicated phase

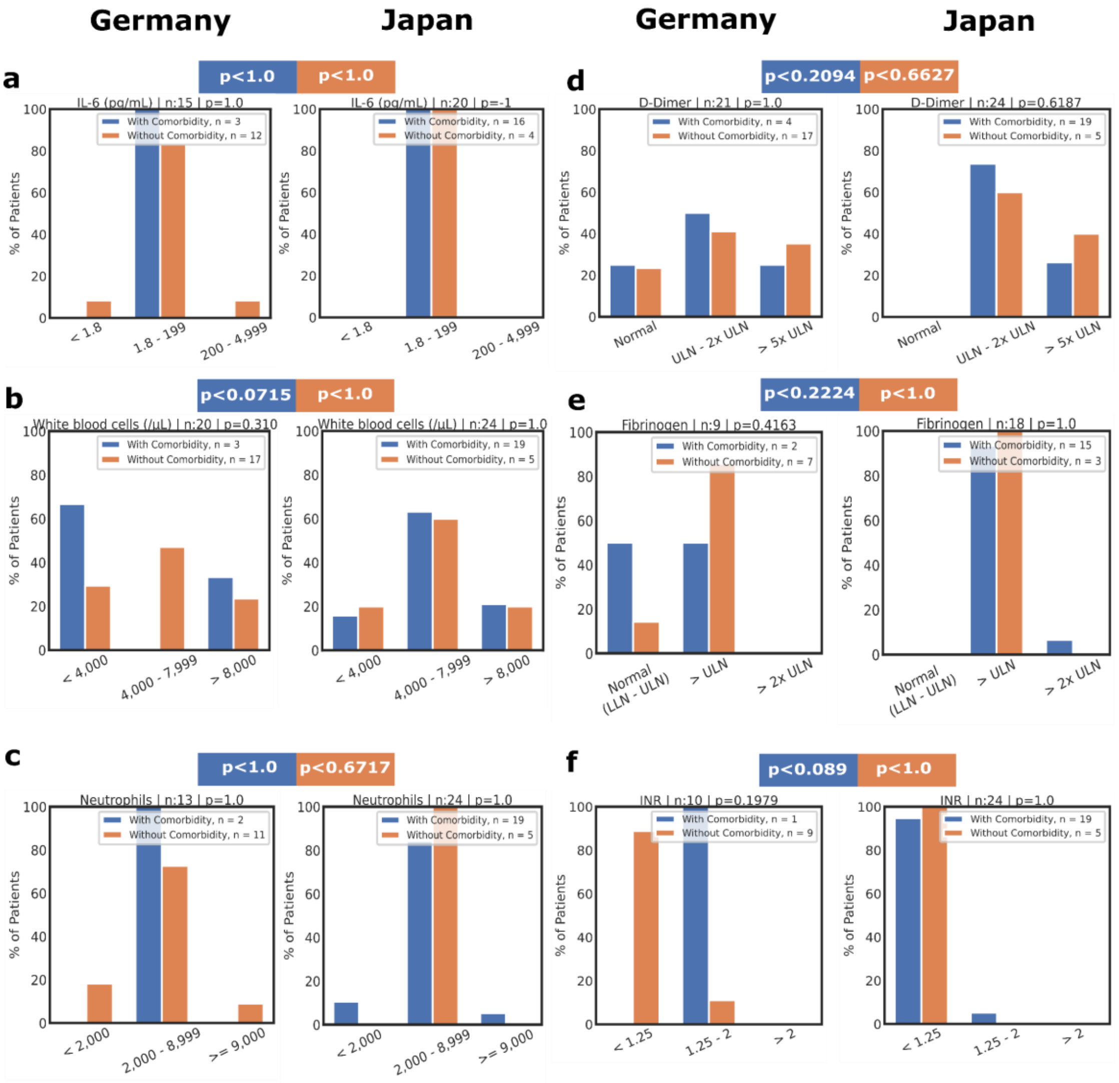

g Uncomplicated, without cardiovascular disease

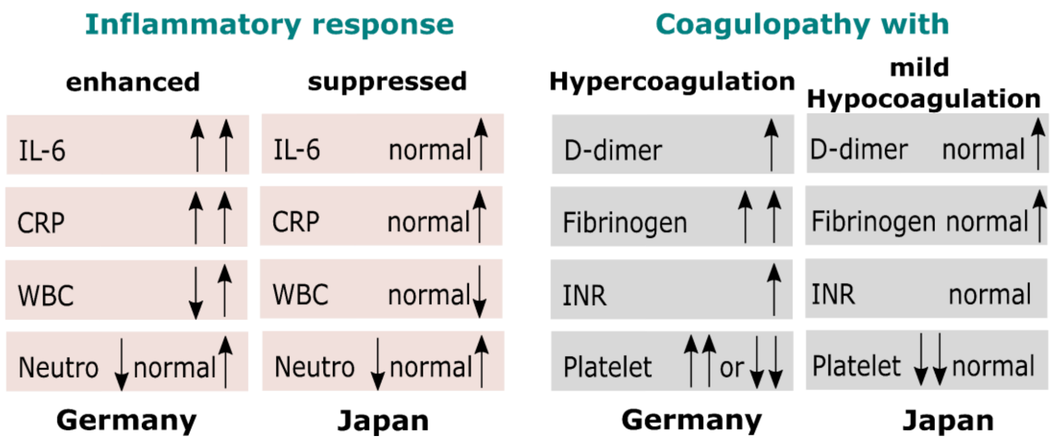

h

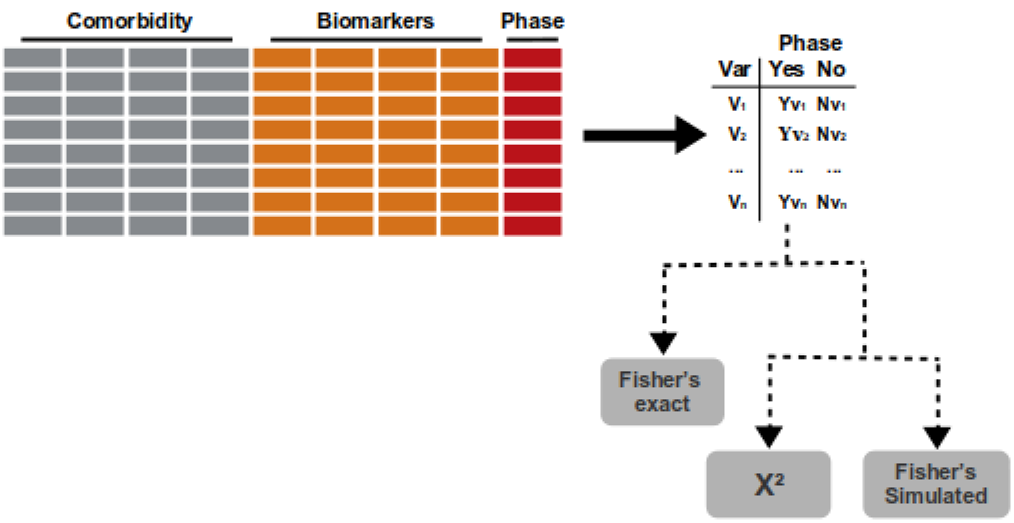

Supplementary Figure S2. Biomarker comparison of propensity score-matched pairs of German and Japanese patients in the complicated phase. (a-f) Biomarker comparison between Japan and Germany in COVID-19 patients in the complicated phase, whereby a patient was considered without cardiovascular comorbidity when no, but only non-cardiovasclUar comorbidities could have been recorded. Serum levels of IL-6 (a), CRP (b), or neutrophil (c) counts in patients at diagnosis from Germany and Japan. Coagulation-associated parameters included levels of D-dimers (d), fibrinogen (e), and INR (f) in patients from Germany and Japan who presented in indicated phase at diagnosis. Patients in the complicated and critical phases were combined and summarized as complicated phases. P values were determined by X2 or Fischer's exact test. P<0.05 were regarded as significant. (g) Differential inflammation- and coagulation-associated responses of German and Japanese COVID-19 patients in the uncomplicated phase at diagnosis. Hyperinflammatory response and coagulopathy with hypercoagulation characterize patients from Germany in the uncomplicated phase. In contrast, patients in the uncomplicated phase in Japan show a suppressed inflammatory response and coagulopathy with hypocoagulation. (h) Scheme of the data analysis. Comorbidities and biomarkers were organized as categorical variables (yes versus no) before predicting the patients' clinical phases within the whole population. The prediction analysis was performed by considering the clinical phase and estimated independence between variables using the indicated experimental setup.
